# Healthcare coverage affects survival of *EGFR-mutant* Thai lung cancer patients

**DOI:** 10.3389/fonc.2023.1047644

**Published:** 2023-02-21

**Authors:** Khantong Khiewngam, Songporn Oranratnachai, Kaettipong Kamprerasart, Patratorn Kunakorntham, Pimtip Sanvarinda, Narumol Trachu, Pongput Pimsa, Jirapath Wiwitkeyoonwong, Thanaporn Thamrongjirapat, Thitiya Dejthevaporn, Ekaphop Sirachainan, Thanyanan Reungwetwattana

**Affiliations:** ^1^ Department of Medicine, Faculty of Medicine Ramathibodi Hospital, Mahidol University, Bangkok, Thailand; ^2^ Division of Medical Oncology, Department of Medicine, Faculty of Medicine Ramathibodi Hospital, Mahidol University, Bangkok, Thailand; ^3^ Faculty of Medicine, Oncology Clinic, Sriphat Medical Center Chiang Mai University, Chiang Mai, Thailand; ^4^ Department of Pathology, Faculty of Medicine Ramathibodi Hospital, Mahidol University, Bangkok, Thailand; ^5^ Data Health for Analysis Unit, Informatics Section, Faculty of Medicine Ramathibodi Hospital, Mahidol University, Bangkok, Thailand; ^6^ Department of Pharmacology, Faculty of Science, Mahidol University, Bangkok, Thailand; ^7^ Research Center, Faculty of Medicine Ramathibodi Hospital, Mahidol University, Bangkok, Thailand; ^8^ Department of Internal Medicine, Sunpasitthiprasong Hospital, Ubon Ratchathani, Thailand; ^9^ Department of Internal Medicine, Police General Hospital, Bangkok, Thailand; ^10^ Ramathibodi Lung Cancer Consortium, Faculty of Medicine Ramathibodi Hospital, Mahidol University, Bangkok, Thailand

**Keywords:** EGFR-TKI, non-small cell lung cancer, drug reimbursement, targeted therapy, Thailand, healthcare coverage

## Abstract

**Background:**

Despite significant benefits of epidermal growth factor receptor-tyrosine kinase inhibitor (EGFR-TKI) treatment in patients with *EGFR*-mutated NSCLC, access remains limited in Thailand and elsewhere.

**Methods:**

Retrospective analysis of patients with locally advanced/recurrent NSCLC and known *EGFR* mutation (*EGFR*m) status treated at Ramathibodi Hospital (2012–2017). Prognostic factors for overall survival (OS), including treatment type and healthcare coverage, were analyzed using Cox regression.

**Results:**

Of 750 patients, 56.3% were *EGFR*m-positive. After first-line therapy (n=646), 29.4% received no subsequent (second-line) treatment. EGFR-TKI-treated *EGFR*m-positive patients survived significantly longer than *EGFR*m-negative patients without EGFR-TKIs (median OS [mOS] 36.4 vs. 11.9 months; hazard ratio HR=0.38 [95%CI 0.32–0.46], *P*<0.001). Cox regression indicated significantly longer OS in patients with comprehensive healthcare coverage that included reimbursement of EGFR-TKIs, versus basic coverage (mOS 27.2 vs. 18.3 months; adjusted HR=0.73 [95%CI 0.59–0.90]). Compared with best supportive care (BSC; reference), EGFR-TKI-treated patients survived significantly longer (mOS 36.5 months; adjusted HR (aHR)=0.26 [95%CI 0.19–0.34]), and versus chemotherapy alone (14.5 months; aHR=0.60 [95%CI 0.47–0.78]). In *EGFR*m-positive patients (n=422), relative survival benefit of EGFR-TKI treatment remained highly significant (aHR[EGFR-TKI]=0.19 [95%CI 0.12–0.29]; aHR(chemotherapy only)=0.50 [95%CI 0.30–0.85]; reference:BSC), indicating that healthcare coverage (reimbursement) affected treatment choice and survival.

**Conclusion:**

Our analysis describes *EGFR*m prevalence and survival benefit of EGFR-TKI therapy for *EGFR*m-positive NSCLC patients treated from 2012–2017, one of the largest such Thai datasets. Together with research by others, these findings contributed evidence supporting the decision to broaden erlotinib access on healthcare schemes in Thailand from 2021, demonstrating the value of local real-world outcome data for healthcare policy decision-making.

## Introduction

Cancer is the leading cause of death, and lung cancer is the second most diagnosed cancer, and cause of cancer deaths after liver cancer in Thailand (1, 2). The NSCLC treatment landscape has evolved with the clinical development and approval of molecular-targeted therapies for patients with specific molecular features, notably epidermal growth factor receptor-tyrosine kinase inhibitors (EGFR-TKI), accompanied by *EGFR* mutation testing (3). The results have been encouraging and are of particular importance in Asian countries as 76% of all activating *EGFR* mutations (*EGFR*m) are detected in Asian patients, with southern Asian patients showing the highest *EGFR*m frequencies (46–52%) (4, 5). In Thailand, *EGFR* mutations were detected in 57–68% of lung adenocarcinoma patients, and the most common mutations were also exon 19 and exon 21 (L858R) point mutation (6, 7). A meta-analysis of seven clinical trials reported prolonged PFS in patients with advanced-stage *EGFR*m-positive NSCLC treated with 1^st^ generation EGFR-TKIs versus chemotherapy, with the greatest benefit observed in patients with exon 19 mutations (8). First-line treatment with 2^nd^ and 3^rd^ generation EGFR-TKI, was associated with significantly longer median PFS compared with 1^st^ generation EGFR-TKI in patients with common sensitizing *EGFR* mutations (9, 10). Currently, the longest OS of *EGFR* mutant lung cancer patients treated by single agent EGFR-TKI is 38.6 month from the FLAURA study which proved the concept and the clinical benefit of EGFR-TKI as the first-line treatment (11).

Despite evidence for the benefits of prescribing EGFR-TKIs as first-line treatment, patient access to EGFR-TKIs in Southeast Asia remains limited. Even though EGFR-TKIs (erlotinib, gefitinib and afatinib) were assessed to have considerable clinical benefit, subsidies or reimbursement for these agents are limited in several Southeast Asian countries, including Myanmar (afatinib unavailable), Laos, and Cambodia as of 2015 (12). Some exceptions included Indonesia that fully subsidized erlotinib, and Vietnam that offered a subsidy of up to 75% for its citizens (12).

In Thailand, access to EGR-TKIs, defined by both costs and availability, has also been limited to varying degrees under the existing healthcare coverage schemes. The three public health insurance schemes in Thailand are the Civil Servant Medical Benefit Scheme (CSMBS; started in 1975), the Social Security Scheme (SSS; started in 1990), and Universal Coverage (UC; started in 2002). The CSMBS and SSS insure individuals employed in the government and private sectors respectively, whereas the UC scheme covers individuals not eligible for the CSMBS or SSS (13, 14).

A medication included in the National List of Essential Medicines (NLEM) is reimbursable for the specified indication under all three healthcare schemes. In the case of certain high-cost drugs, including molecular targeted drugs, these have been reimbursable only for patients with CSMBS coverage, under the Oncology Prior Authorization Program (OCPA). Since 2006, CSMBS-insured patients could reimburse gefitinib and erlotinib for third-line treatment of NSCLC under the OCPA (no *EGFR* mutation testing required). From 2018 onwards, under the OCPA, CSMBS-insured individuals could reimburse gefitinib as first-line treatment (*EGFR*m-positive patients only), and in 2019 osimertinib as second- or third-line treatment (*T790M*-positive patients after 1^st^ generation EGFR-TKI treatment failure). Prior to December 2020, individuals with only UC or SSS coverage could not receive reimbursement for EGFR-TKI treatment (any line).

The decision to include a medication into the NLEM is made based on the Thai Health Technology Assessment guidelines, which evaluate the benefits of the medication based on available data on costs and health outcomes (12, 15). To help national healthcare policy-makers in Thailand and other Southeast Asian countries make informed and up-to-date decisions that affect cancer care, it is highly important that treatment outcomes in real-life practice with important medicines, such as EGFR-TKIs, are explored and well documented. Our study analyzed treatment outcomes for NSCLC patients in Thailand, particularly real-world clinical benefit of EGFR-TKI therapy for *EGFR*m-positive NSCLC patients at the time of EGFR-TKI could not reimburse for UC and SSS patients in Thailand. This data was contributing to a body of data essential for evaluation and improvement of EGFR-TKI reimbursement programs in Thailand. We hope this real-world evidence could provide the useful data for helping improvement of EGFR TKI reimbursement policy in the other developing countries as well.

## Patients and methods

### Study participants and data collection

This retrospective study included patients with locally advanced/recurrent NSCLC treated at Ramathibodi hospital from 9 May 2012 to 30 April 2017, and who had known *EGFR* mutation status (tissue test). Patients with early-stage NSCLC (stage I, II or IIIA), insufficient medical data, or those found to have non-*EGFR* driver mutations were excluded. The Human Research Ethics Committee of the Ramathibodi Hospital approved the study (IRB No. MURA2020/304) and waived the requirement for informed patient consent. Clinical data from the time of diagnosis to time of death were obtained from electronic database records.

**Figure 1:**
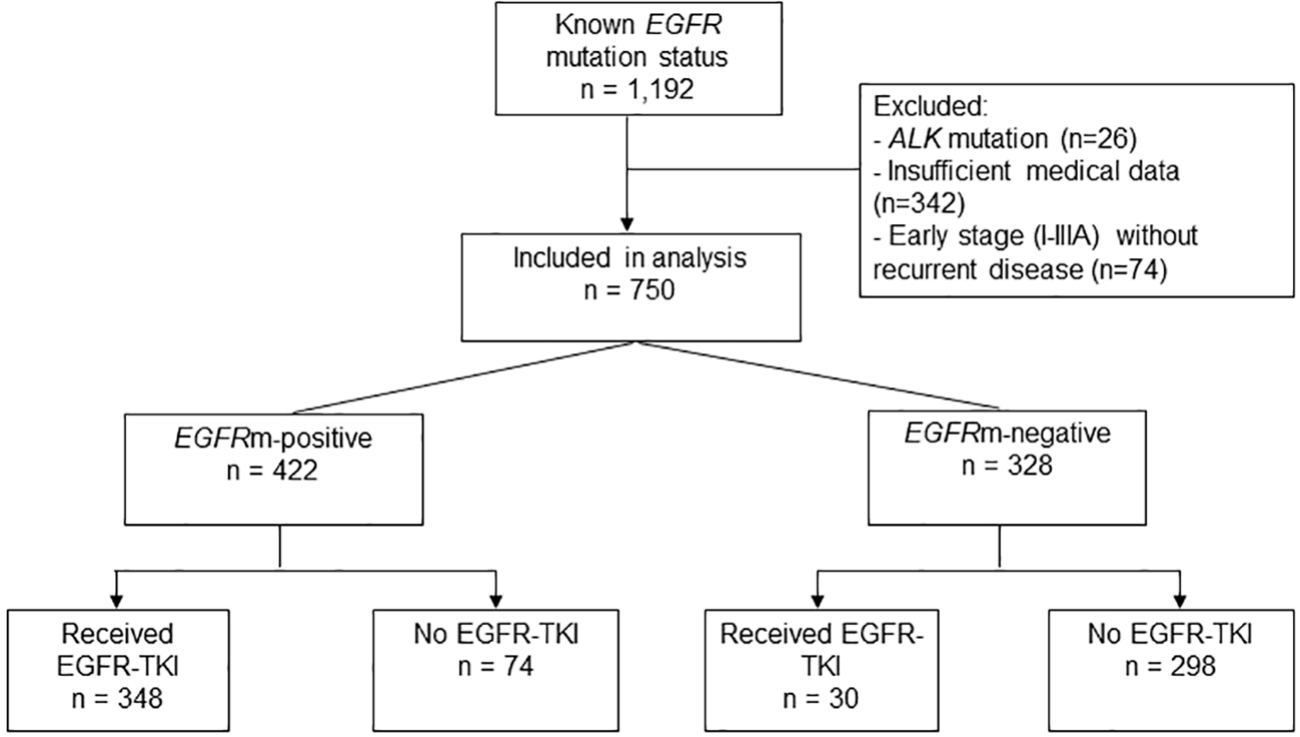
Flowchart of patient selection in the study.

Patients were categorized into four groups based on their *EGFR* mutation status and type of treatment received (**Figure 1**). Mutations were categorized as: Common *EGFR* activating mutations including exon 19 (*del19*) or *L858R*; uncommon activating mutations including *G719X, L861Q, del19* + *L858R*, *del19* + *S768I, L858R* + *S768I*, *G719X*+*S768I*; and uncommon resistance mutations including exon 20 insertions (*20ins*), *del19+T790M*, *L858R+T790M*, *L858R+20ins* and *L861Q+T790M.*

### Outcomes

Overall survival (OS) was defined as the time from diagnosis of advanced-stage disease to death from any causes, or the end of the data collection period (November 30, 2019). Time to treatment failure (TTF) of EGFR-TKI was defined as the time from initiation of EGFR-TKI treatment to the time of stopping EGFR-TKI.

### Statistical analysis

Patient characteristics were summarized using descriptive statistical techniques. Chi-square or Fisher’s exact tests were used to analyze association between categorical variables. The Kaplan-Meier method was used to estimate survival probabilities over time. Univariate and multivariate Cox proportional hazards regression models were used to analyze relationships between prognostic factors and survival outcomes. All statistical analyses were performed using Stata software (version 15). Significance tests were two-sided, and performed at the 5% significance level (α=0.05).

## Results

### Patient characteristics

Of 1,192 NSCLC patients with available *EGFR* mutation test results diagnosed/treated at our institution from May 2012 to April 2017, 442 patients (37.1%) were excluded from the analysis due to insufficient medical data (28.7%), presence of *ALK* mutations (2.2%), or stage I–IIIA at diagnosis without recurrent disease (6.2%) (**Figure 1**). The final analysis population included 750 patients with locally advanced, advanced, or recurrence (stage I-III at diagnosis with recurrent disease), and known *EGFR* mutation status (**Table 1**; **Figure 1**).

**Table 1:** Demographic characteristics.

Characteristics	All patients N = 750	*EGFR*m+ N = 422	*EGFR*m− N = 328	*P* ^†^
Age (mean ± SD) years	64.5 ± 11.4	64.0 ± 11.1	64.5 ± 11.8	0.596
Gender				<0.001
Male	343 (45.7)	150 (35.5)	193 (58.8)	
Female	407 (54.3)	272 (64.5)	135 (41.2)	
Histology subtype				<0.001
Adenocarcinoma	624 (83.2)	377 (89.3)	247 (75.3)	
Adenosquamous carcinoma	14 (1.9)	6 (1.4)	8 (2.4)	
Squamous cell carcinoma	17 (2.3)	5 (1.2)	12 (3.7)	
Other NSCLC/unknown	95 (12.7)	34 (8.1)	61 (18.6)	
Stage at diagnosis				0.043
I	58 (7.7)	39 (9.2)	19 (5.8)	
II	50 (6.7)	26 (6.2)	24 (7.3)	
III	89 (11.9)	40 (9.5)	49 (15.0)	
IV	553 (73.7)	317 (75.1)	236 (71.9)	
Smoking status				<0.001
Current/Ex-smoker	277 (36.9)	104 (24.6)	173 (52.7)	
Never-smoker	473 (63.1)	318 (75.4)	155 (47.3)	
Healthcare coverage status				0.567
UC/SSS	167 (22.3)	89 (21.1)	78 (23.8)	
CSMBS/SE	405 (54.0)	228 (54.0)	177 (54.0)	
Self-pay	178 (23.7)	105 (24.9)	73 (22.2)	
Lines of treatment received				<0.001
No treatment	104 (13.9)	33 (7.8)	71 (21.7)	
1	192 (25.6)	93 (22.1)	99 (30.2)	
2	176 (23.5)	109 (25.8)	67 (20.4)	
≥3	278 (37.1)	187 (44.3)	91 (27.7)	
EGFR-TKIs received	N = 378	N = 348	N= 30	0.009
1 agent	229 (60.6)	203 (58.3)	26 (86.7)	
2 agents	127 (33.6)	123 (35.4)	4 (13.3)	
≥3 agents	22 (5.8)	22 (6.3)	0 (0)	

n (%) unless otherwise stated.

**^†^** P-value from chi-squared or Fisher’s exact test. P-values <0.05 were considered statistically significant.

UC, Universal Coverage; SSS, Social Security Scheme; CSMBS, Civil Servant Medical Benefit Scheme; SE, State Enterprise Scheme; EGFR-TKI, Epidermal growth factor receptor-tyrosine kinase inhibitor; EGFRm+, positive for EGFR activating mutation, EGFRm−, wild-type EGFR; SD, standard deviation.

For the total NSCLC patient population (n=750), the median follow-up time was 24.9 months (range, 22.0–27.5) and the median follow-up time for patients diagnosed with stage IV NSCLC was 21.4 months (range, 19.6–23.4). Half of the patients (54.3%) were female, and the majority had adenocarcinoma histology (83.2%) (**Table 1**). Slightly over half of all patients (54.0%) had CSMBS or state enterprise (CSMBS/SE) healthcare coverage, 22.3% had UC/SSS healthcare coverage, and 23.7% were self-paying. The profile of healthcare coverage status was similar in the *EGFR*m-positive and *EGFR*m-negative groups (**Table 1**).

Over half of the patients (56.3%; n=422) were *EGFR*m-positive. *EGFR*m-positive patients were predominantly never-smokers (75.4%) and female (64.5%). Most patients had common *EGFR* activating mutations (55.2% with exon 19 deletion; 32.7% with L858R point mutation), and 12.1% had double mutations or uncommon mutations (**Supplementary Table A**).

### Systemic therapy

Within the overall NSCLC patient population (n=750), most patients (58.4%) received chemotherapy as their first-line treatment, mainly platinum-doublet regimens (**Supplementary Table B**). Two-hundred and eight patients (27.7%) received first-line EGFR-TKI therapy, and 13.9% did not receive any systemic therapy. Sixty-three patients received erlotinib, 111 patients received gefitinib, 20 patients received afatinib, 7 patients received osimertinib, and the other 7 patients received EGFR-TKI in clinical trial as the first-line treatment (**Supplement Table B**). There was a 29.4% drop-off rate from first-line to second-line treatment, and a 38.4% drop-off rate from second-line to third-line treatment (**Supplementary Table B**).

Among *EGFR*m-positive patients (n=422), 109 patients (25.8%) received two lines of treatment, and 187 (44.3%) received three or more lines of treatment, including chemotherapy. The majority (58.3%) of *EGFR*m-positive patients received only one type of EGFR-TKI (**Table 1**).

### Overall survival according to EGFRm status and EGFR-TKI treatment

**Figure 2:**
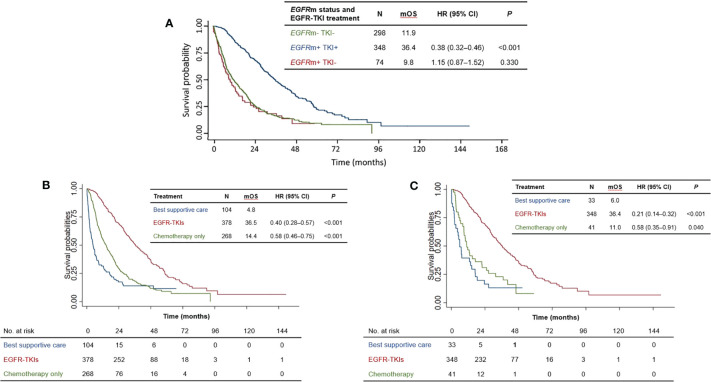
**(A)** Overall survival of patients according to *EGFR*m status and EGFR-TKI treatment. **(B)** Overall survival of patients according to treatment type for the total patient population. **(C)** Overall survival of patients according to treatment type for *EGFR*m-positive patients. mOS = median overall survival; HR, hazard ratio; CI, confidence interval; EGFR-TKI, Epidermal growth factor receptor-tyrosine kinase inhibitor; *EGFR*m+, positive for EGFR activating mutation, *EGFR*m−, wild-type EGFR.

Since EGFR-TKI treatment is indicated specifically for NSCLC patients with activating *EGFR* mutations, we first investigated the influence of EGFR-TKI treatment on survival. *EGFR*m-positive patients treated with EGFR-TKIs survived significantly longer than the reference group of *EGFR*m-negative patients not treated with EGFR-TKIs (median OS 36.4 vs. 11.9 months; HR=0.38 [95% CI 0.32–0.46], *P*<0.001) (**Figure 2A**). Survival of *EGFR*m-positive patients who did not receive EGFR-TKI treatment was not significantly different from the reference group (median OS: 9.8 vs. 11.9 months: HR=1.15 [95% CI 0.87–1.52], *P*=0.330).

### Overall survival according to treatment type

One hundred and four patients underwent best supportive care alone due to poor performance status and rapid progression of disease. Compared with best supportive care alone (median OS 4.8 months), EGFR-TKI treatment significantly prolonged survival (median OS 36.5 months, HR=0.40 [95% CI 0.28–0.57, *P*<0.001]) (**Figure 2B**) for the whole population (n=750). Notably, EGFR-TKI-treated patients also survived longer than those who only received chemotherapy, who had a median OS of 14.4 months (HR=0.58 [95% CI: 0.46–0.75, *P*<0.001] versus best supportive care) (**Figure 2B**).

Our analysis of the subset of *EGFR*m-positive patients (n=422) revealed a similar trend (**Figure 2C**). Once again, EGFR-TKI treatment was associated with longer survival than chemotherapy alone (median OS: 36.4 months and 11.0 months, respectively), with non-overlapping 95% CIs of their hazard ratios versus best supportive care: HR(EGFR-TKI)=0.21 [95% CI: 0.14–0.32], HR(chemotherapy)=0.58 [95% CI: 0.35–0.91]. Taken together, these results indicate that appropriate EGFR-TKI treatment according to mutation status (i.e., for *EGFR*m-positive patients) significantly prolonged overall survival, compared with chemotherapy alone or best supportive care.

### Overall survival according to healthcare coverage scheme

**Figure 3:**
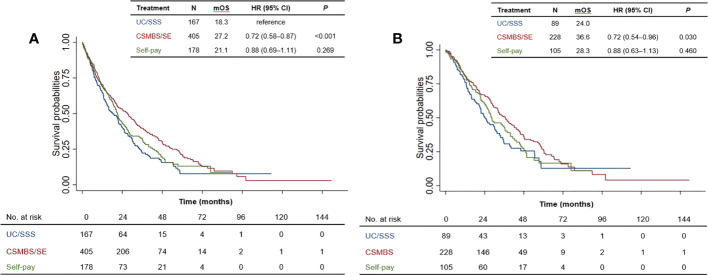
Overall survival of patients according to healthcare coverage scheme for **(A)** total patient population, and **(B)** *EGFR*m-positive patients. mOS, median overall survival; HR, hazard ratio; CI, confidence interval. UC, Universal Coverage; SSS, Social Security Scheme; CSMBS, Civil Servant Medical Benefit Scheme; SE, State Enterprise Scheme.

Since healthcare coverage directly affects drug reimbursement and access to treatments such as EGFR-TKIs, we next investigated whether healthcare coverage status was related to survival outcomes. Among patients with more comprehensive coverage (CSMBS/SE), median OS was significantly longer than for those with basic coverage (UC/SSS): median OS 27.2 versus 18.3 months, HR=0.72 [95% CI 0.58–0.88], *P*<0.001 (**Figure 3A**). Overall survival among self-paying patients was not significantly different from those with only UC/SSS healthcare coverage. Similarly, among *EGFR*m-positive patients, CSMBS/SE patients showed longer significantly longer survival than UC/SSS patients: median OS 36.6 versus 24.0 months, HR=0.72 [95% CI 0.54–0.96], *P*=0.030 (**Figure 3B**). In both the total patient population and in the *EGFR*m-positive subset, having more comprehensive healthcare coverage (CSMBS/SE) was associated with significantly longer survival than basic UC/SSS coverage.

### Clinical outcomes in EGFR-TKI-treated patients with different EGFR mutation subtypes

Among patients with *EGFR* activating mutations who were treated with EGFR-TKIs, median OS was 35 months or longer, and did not differ significantly across mutation subtypes (**Supplementary Figure A**). Time to EGFR-TKI treatment failure was similar in patients with common *EGFR* activating mutations only (approximately 13 months for those with exon 19 deletion or L858R), but significantly shorter in those who also had the T790M resistance mutation (T790M+: 7.6 months) (**Supplementary Table C**).

In the subset of T790M+ patients, treatment with osimertinib significantly prolonged survival: median OS was 57.4 months with osimertinib treatment versus 25.2 months without osimertinib (HR=0.24 [95% CI 0.13–0.44, p<0.001]) (**Supplementary Figure B**).

### Cox regression analysis of prognostic factors for survival

To study the range of factors that potentially impact the survival of NSCLC patients, we performed multivariate Cox regression to analyze prognostic factors for survival in the total NSCLC patient population, and in the subset of *EGFR*m-positive patients.

In the total NSCLC patient population (n=750), potentially significant prognostic variables identified from univariate analyses included age, gender, smoking status, type of healthcare coverage, *EGFR*m status and treatment type. Of these, only age, gender, healthcare coverage type and treatment type remained statistically significantly related to OS in multivariate analyses (**Table 2**). The largest survival benefit was observed in patients who were female (adjusted HR=0.79 [95% CI: 0.63–0.99]), those who had CSMBS/SE healthcare coverage (adjusted HR=0.73 [95% CI: 0.59–0.90] versus UC/SSS), or were treated with EGFR-TKIs (adjusted HR=0.26 [95% CI: 0.19–0.34] versus best supportive care only).

**Table 2:** Cox regression analysis of prognostic factors associated with overall survival of all patients.

Prognostic factors	N	Overall survival (months)	Univariate analysis		Multivariate analysis	
			HR (95% CI)	*P* ^†^	Adjusted HR (95% CI)	*P* ^†^
Age	750		1.01 (1.00–1.02)	0.004*	1.01 (1.00–1.02)	<0.001*
Gender						
Male	343	17.1	1		1	
Female	228	28.5	0.67 (0.57–0.79)	<0.001*	0.79 (0.63–0.99)	0.041*
Smoking status						
Current/Ex-smoker	277	14.6	1		1	
Never-smoker	473	27.8	0.64 (0.55–0.77)	<0.001*	0.99 (0.79–1.25)	0.950
Healthcare coverage status						
UC/SSS	167	18.3	1		1	
CSMBS/SE	405	27.2	0.72 (0.58–0.87)	<0.001*	0.73 (0.59–0.90)	0.003*
Self-pay	178	21.1	0.88 (0.69–1.11)	0.269	1.00 (0.79–1.27)	0.990
*EGFR* mutation						
No	328	13.8	1		1	
Yes	422	32.0	0.51 (0.43–0.60)	<0.001*	1.03 (0.81–1.32)	0.790
Treatment						
Best supportive care	104	4.8	1		1	
Chemotherapy alone	268	14.5	0.58 (0.46–0.75)	<0.001*	0.60 (0.47–0.78)	<0.001*
EGFR-TKI treatment	378	36.5	0.40 (0.28–0.57)	<0.001*	0.26 (0.19–0.34)	<0.001*

**^†^** P-values calculated from Cox proportional hazards model.

^*^P-values <0.05 were considered statistically significant.

UC, Universal Coverage; SSS, Social Security Scheme; CSMBS, Civil Servant Medical Benefit Scheme; SE, State Enterprise Scheme; EGFR-TKI, Epidermal growth factor receptor-tyrosine kinase inhibitors; CI, confidence interval; HR, hazard ratio.

In the subset of *EGFR*m-positive patients (n=422), the potentially significant prognostic variables identified from univariate analyses were gender, smoking status, healthcare coverage status and treatment type. Of these, only gender and treatment type remained significant prognostic factors for OS in multivariate analyses (**Table 3**). The CSMBS/SE group also showed a trend toward longer OS compared with the UC/SSS or self-paying groups in multivariate analyses (**Table 3**). As observed in the total NSCLC patient population, survival with EGFR-TKI treatment in *EGFR*m-positive patients was also longer than with chemotherapy alone (adjusted HR[EGFR-TKI]=0.19 [95% CI: 0.12–0.29], adjusted HR (chemotherapy)=0.50 [95% CI: 0.30–0.85]), and both significantly prolonged OS compared with best supportive care.

**Table 3:** Cox regression analysis of prognostic factors associated with overall survival in *EGFR*m-positive patients.

Prognostic factors	N	Overall survival (months)	Univariate analysis		Multivariate analysis	
			HR (95% CI)	*P* ^†^	Adjusted HR (95% CI)	*P* ^†^
Age	422		1.00 (0.99–1.01)	0.740	1.00 (0.99–1.01)	0.960
Gender						
Male	150	25.8	1		1	
Female	272	35.8	0.74 (0.59–0.94)	0.010*	0.70 (0.52–0.95)	0.021*
Smoking status						
Current/Ex-smoker	104	24.4	1		1	
Never-smoker	318	34.9	0.75 (0.58–0.97)	0.030*	0.96 (0.68–1.34)	0.790
Healthcare coverage status						
UC/SSS	89	24.0	1		1	
CSMBS/SE	228	36.6	0.72 (0.54–0.96)	0.030*	0.76 (0.56–1.03)	0.070
Self-pay	105	28.3	0.88 (0.63–1.13)	0.460	1.01 (0.72–1.42)	0.950
Treatment						
Best supportive care	33	6.0	1		1	
Chemotherapy alone	41	11.0	0.58 (0.35–0.91)	0.040*	0.50 (0.30–0.85)	0.010*
EGFR-TKI treatment	348	36.4	0.21 (0.14–0.32)	<0.001*	0.19 (0.12–0.29)	<0.001*

**^†^** P-values calculated from Cox proportional hazards model.

^*^P-values <0.05 were considered statistically significant.

UC, Universal Coverage; SSS, Social Security Scheme; CSMBS, Civil Servant Medical Benefit Scheme; SE, State Enterprise Scheme; EGFR-TKI, Epidermal growth factor receptor-tyrosine kinase inhibitor; CI, confidence interval; HR, hazard ratio.

These results indicate that the observed influence of healthcare coverage status on survival can be attributed to drug reimbursement and access to EGFR-TKI therapy, especially for *EGFRm*-positive patients.

## Discussion

The clinical development of EGFR-TKIs offered a more efficacious and tolerable alternative to standard cytotoxic chemotherapy for patients with *EGFR*-mutated lung cancer, and this has profoundly altered the NSCLC treatment landscape in the past decade. Like other molecular targeted therapies, EGFR-TKIs have the potential to improve clinical outcomes for large numbers of NSCLC patients in Asia and other regions where the prevalence of actionable molecular alterations is high (4, 5). Studies suggest that *EGFR* mutations are present in ≥40–68% of Thai NSCLC patients, with higher frequencies among those with adenocarcinoma histology (6, 7, 16).

Clinical trials provide evidence of significant benefit with 1^st^/2^nd^-generation EGFR-TKIs in early-line treatment of *EGFR*m-positive NSCLC (8–11, 17, 18), additionally, these agents are recommended in numerous clinical practice guidelines and are included in the WHO Essential Medicines List (19). Even so, reimbursement and access to EGFR-TKI therapy remain limited in a number of countries, even where these agents have been approved by national health authorities for treating advanced-stage NSCLC. Examples include reimbursement of selected agents only under certain healthcare schemes, and/or only after failure of multiple lines of other therapy, as was the case in Thailand for 1^st^ generation EGFR-TKIs prior to 2021.

Moreover, in real-world practice, a substantial proportion of patients diagnosed with advanced NSCLC remain untreated, or receive only limited therapy. Due to factors such as rapid disease progression, decline in PS, and/or toxicity from previous therapy, high drop-off rates after first-line therapy (≥20–30% or more with successive lines) have been reported in a number of countries (20–24). We noted similar trends in our analysis, with drop-off rates of 29% and 38% after first-line and second-line therapy, respectively; in fact, 14% of our patients received no active anticancer treatment at all (supportive care only). Cost barriers and limited access to superior agents for early-line therapy may exacerbate such problems with under-treatment.

In many regions, the real-world impact of limited or differential access on clinical outcomes has not been well quantified, potentially hindering national-level decision-making that could improve cancer care. Our analysis of real-world treatment patterns and outcomes in Thai NSCLC patients (2012–2017) was the largest to date for this time period, and was significant because it highlighted that patients’ survival was significantly associated with their healthcare coverage status and especially the type of treatment received. Specifically, for *EGFR*m-positive patients, receiving EGFR-TKI therapy (reimbursable only under the more comprehensive CSMBS/SE schemes) was associated with longer survival than chemotherapy alone or best supportive care. Our results showed that, for *EGFR*m-positive NSCLC patients who received EGFR-TKI treatment, real-world clinical outcomes (median OS approximately 35 months, median TTF approximately 12 months) were comparable with those reported in other countries (20, 21, 23, 25, 26). However, some of the clinical factors that might affect the survival of *EGFR*m-positive patients such as performance status, brain metastases, and post EGFR-TKI treatment were not retrieved from our database. This is one of the limitations of this report. In contrast, *EGFR*m-negative NSCLC patients (who are not considered to benefit from EGFR-TKI therapy), our analysis suggested a possibility that choice of treatment based on healthcare coverage status may also influence survival, and this possibility may need to be explored in future work. For example, pemetrexed and vinorelbine could not reimburse in UC and SSS patients which might affect the survival of patients. Although not explored in the present analysis, the influence of healthcare coverage on *EGFR* mutation testing practice is a related issue that also warrants investigation.

Along with others, these findings on the clinical benefit of EGFR-TKI treatment contributed real-world evidence to support re-evaluation of EGFR-TKI reimbursement. In 2020, with the combined efforts and cooperation of other oncologists and healthcare policy-makers, a decision was reached to include erlotinib (generic) in the Thai NLEM as a first-line treatment for patients with advanced *EGFR*m-positive NSCLC from 2021 onwards (27). This potentially broadens access to EGFR-TKI first-line therapy on all healthcare schemes in Thailand. Following the update, the CSMBS scheme now reflects the recognition of erlotinib as the preferred first-line EGFR-TKI, in line with other national healthcare schemes. For CSMBS-insured individuals, reimbursement of gefitinib can still be requested under the OCPA if the patient is unable to tolerate the side effects of erlotinib first-line therapy. However, given that over two-thirds of the population (72.2%) are only covered by the UC scheme, many patients could still have limited access to 2^nd^ and 3^rd^ generation EGFR-TKI therapies such as afatinib, dacomitinib, and osimertinib. Only CSMBS patients could reimburse osimertinib as the second-line treatment in *T790M*-positive patients.

The landscape of lung cancer treatment continues to evolve rapidly. Currently, there was a study (ADUARA) significantly demonstrated increasing of disease-free survival (DFS) of 3-year osimertinib in adjuvant treatment for stage IB – stage IIIA *EGFR*m-positive patients. This indication of osimertinib also approved by Thai FDA, but the patients could not reimburse from all healthcare schemes. Therefore, it will be important to continue generating high-quality data on the local impact of treatments, to support national healthcare policy-makers in timely evaluation and up-to-date decisions on first-line treatments in metastatic disease or adjuvant treatment in early stage disease, their indications and extent of subsidies.

## Data availability statement

The raw data supporting the conclusions of this article will be made available by the authors, without undue reservation.

## Ethics statement

The studies involving human participants were reviewed and approved by Human Research Ethics Committee, Faculty of Medicine Ramathibodi Hospital, Mahidol University COA. MURA2020/304. Written informed consent for participation was not required for this study in accordance with the national legislation and the institutional requirements.

## Author contributions

KKh : Investigation, methodology, patient enrollment, data curation, validation, data interpretation, project administration, resources, formal analysis, original draft. SO: Investigation, methodology, software, validation, formal analysis, review & edit manuscript. KKa : Investigation, patient enrollment, data curation, review & edit manuscript. PK: patient enrollment, data curation. PS: Investigation, patient enrollment, review & edit manuscript. NT: investigation, patient enrollment, review & edit manuscript. PP: Patient enrollment, data curation, review & edit manuscript. JW: patient enrollment, data curation, review & edit manuscript. TT: Visualization, patient enrollment, data curation, review & edit manuscript. TD: Study design, conceptualization, visualization, patient enrollment, data analysis, data interpretation, conclusion, suggestion, review & edit manuscript. ES: Visualization, data curation, review & edit manuscript. TR: Supervision, study design, conceptualization, visualization, investigation, methodology, analysis, data curation, validation, conclusion, data interpretation, original draft, writing - review & editing. All authors contributed to the article and approved the submitted version.

## Supplementary material

Supplementary Figure A Overall survival by *EGFR* mutation subtype. mOS = median overall survival; HR = hazard ratio; CI = confidence interval.

Supplementary Figure B Overall survival in patients with acquired T790M mutation with and without osimertinib treatment. mOS = median overall survival; HR = hazard ratio; CI = confidence interval.

## References

[1] Reungwetwattana T Oranratnachai S Puataweepong P Tangsujaritvijit V Cherntanomwong P. Lung cancer in Thailand. J Thorac Oncol (2020) 15:1714–21. doi: 10.1016/j.jtho.2020.04.024 33148410

[2] International Agency for Research on Cancer. The global cancer observatory: Thailand (Source: Globalcan 2020)(2021). Available from: https://gco.iarc.fr/today/data/factsheets/populations/764-thailand-fact-sheets.pdf

[3] Hill A Gupta R Zhao D Vankina R Amanam I Salgia R. Targeted therapies in non-small-Cell lung cancer. Cancer Treat Res (2019) 178:3–43. doi: 10.1007/978-3-030-16391-4_1 31209840

[4] Graham RP Treece AL Lindeman NI Vasalos P Shan M Jennings LJ. Worldwide frequency of commonly detected EGFR mutations. Arch Pathol Lab Med (2018) 142:163–7. doi: 10.5858/arpa.2016-0579-CP 29106293

[5] Shi Y Au JS Thongprasert S Srinivasan S Tsai Khoa C- MT. A prospective, molecular epidemiology study of EGFR mutations in Asian patients with advanced non-small-cell lung cancer of adenocarcinoma histology (PIONEER). J Thorac Oncol (2014) 9:154–62. doi: 10.1097/JTO.0000000000000033 PMC4132036 24419411

[6] Sriuranpong V Chantranuwat C Huapai N Chalermchai T Leungtaweeboon K Lertsanguansinchai P. High frequency of mutation of epidermal growth factor receptor in lung adenocarcinoma in Thailand. Cancer Lett (2006) 239:292–7. doi: 10.1016/j.canlet.2005.08.029 16243431

[7] Detarkom S Incharoen P Jinawat A Trachu N Kamprerasart K Prasongsook N. P3.09-08 tumor heterogeneity and molecular profile of NSCLC in Thai population. J Thorac Oncol (2018) 13:S949–50. doi: 10.1016/j.jtho.2018.08.1777

[8] Lee CK Wu YL Ding PN Lord SJ Inoue A Zhou C. Impact of specific epidermal growth factor receptor (EGFR) mutations and clinical characteristics on outcomes after treatment with EGFR tyrosine kinase inhibitors versus chemotherapy in EGFR-mutant lung cancer: A meta-analysis. J Clin Oncol (2015) 33:1958–65. doi: 10.1200/JCO.2014.58.1736 25897154

[9] Park K Tan EH O'Byrne K Zhang L Boyer M Mok T. Afatinib versus gefitinib as first-line treatment of patients with EGFR mutation-positive non-small-cell lung cancer (LUX-lung 7): A phase 2B, open-label, randomised controlled trial. Lancet Oncol (2016) 17(5):577–89. doi: 10.1016/S1470-2045(16)30033-X 27083334

[10] Soria JC Ohe Y Vansteenkiste J Reungwetwattana T Chewaskulyong B Lee KH. Osimertinib in untreated EGFR-mutated advanced non-Small-Cell lung cancer. N Engl J Med (2018) 378:113–25. doi: 10.1056/NEJMoa1713137 29151359

[11] Ramalingam SS Vansteenkiste J Planchard D Cho BC Gray JE Ohe Y. Overall survival with osimertinib in untreated, EGFR-mutated advanced NSCLC. N Engl J Med (2020) 382:41–50. doi: 10.1056/NEJMoa1913662 31751012

[12] Eniu A Cherny NI Bertram M Thongprasert S Douillard J Bricalli G. Cancer medicines in Asia and Asia-pacific: What is available, and is it effective enough? ESMO Open (2019) 4:e000483. doi: 10.1136/esmoopen-2018-000483 31423334 PMC6677966

[13] Patikorn C Taychakhoonavudh S Thathong T Anantachoti P. Patient access to anti-cancer medicines under public health insurance schemes in Thailand: A mixed methods study. Thai J Pharm Sci (2019) 43:168–78. Available at: http://www.tjps.pharm.chula.ac.th/ojs/index.php/tjps/article/view/27

[14] Paek SC Meemon N Wan TT. Thailand's universal coverage scheme and its impact on health-seeking behavior. Springerplus. (2016) 5:1952. doi: 10.1186/s40064-016-3665-4 27933235 PMC5104696

[15] Youngkong S. The Thai pharmacoeconomics guidelines and its application in Thailand. Thailand: Mahidol University (2016) p. 1–15. Available at: https://www.ispor.org/docs/default-source/presentations/780.pdf?sfvrsn=d2d32686_1

[16] Chantharasamee J Poungvarin N Danchaivijitr P Techawatanawanna S. Clinical outcome of treatment of metastatic non-small cell lung cancer in patients harboring uncommon EGFR mutation. BMC Cancer. (2019) 19:701. doi: 10.1186/s12885-019-5913-9 31315599 PMC6637469

[17] Ramalingam SS Yang JC Lee CK Kurata T Kim D John T. Osimertinib as first-line treatment of EGFR mutation-positive advanced non-Small-Cell lung cancer. J Clin Oncol (2018) 36:841–9. doi: 10.1200/JCO.2017.74.7576 28841389

[18] Greenhalgh J Boland A Bates V Vecchio F Dundar Y Chaplin M. First-line treatment of advanced epidermal growth factor receptor (EGFR) mutation positive non-squamous non-small cell lung cancer. Cochrane Database Syst Rev (2021) 3:CD010383. doi: 10.1002/14651858.CD010383.pub3 33734432 PMC8092455

[19] World Health Organization. The selection and use of essential medicines: report of the WHO expert committee on selection and use of essential medicines, 2019 (including the 21st WHO model list of essential medicines and the 7th WHO model list of essential medicines for children). Geneva: World Health Organization (2019).

[20] Agulnik JS Kasymjanova G Pepe C Hurry M Walton RN Sakr L. Real-world pattern of treatment and clinical outcomes of EGFR-mutant non-small cell lung cancer in a single academic centre in Quebec. Curr Oncol (2021) 28:5179–91. doi: 10.3390/curroncol28060434 PMC8700535 34940073

[21] Chua B Tan EH Lim DWT Ang MK Tan DSW Ng QS. Real world data on epidermal growth factor receptor (EGFR) tyrosine kinase inhibitors (TKI) use in advanced/metastatic non-small cell lung cancer (NSCLC) with EGFR mutations in Singapore. Ann Oncol (2018) 29:ix161. doi: 10.1093/annonc/mdy425.033

[22] Sacher AG Le LW Lau A Earle CC Leighl NB. Real-world chemotherapy treatment patterns in metastatic non-small cell lung cancer: Are patients undertreated? Cancer. (2015) 121:2562–9. doi: 10.1002/cncr.29386 25891153

[23] Okamoto I Morita S Tashiro N Imamura F Inoue A Seto T. Real world treatment and outcomes in EGFR mutation-positive non-small cell lung cancer: Long-term follow-up of a large patient cohort. Lung Cancer. (2018) 117:14–9. doi: 10.1016/j.lungcan.2018.01.005 29496250

[24] Chiang A Fernandes A Pavilack M Wu J Laliberté F Duh MS. MA15.11 real world biomarker testing and treatment patterns in patients with advanced NSCLC receiving EGFR-TKIs. J Thorac Oncol (2018) 13:S410–1. doi: 10.1016/j.jtho.2018.08.447

[25] Gautam S Herms L Bartolome L Pastel M Wilner K Fisher M. P76.69 real-world effectiveness of EGFR TKI first-line treatment of advanced EGFR mutation-positive non-small cell lung cancer in US. J Thorac Oncol (2021) 16:S618–9. doi: 10.1016/j.jtho.2021.01.1126

[26] Van Luan P Tien ND Hai NM Tien ND Duyen TT. Real-world analysis of the effect of gefitinib as a first-line therapy in patients with advanced non-small cell lung cancer with EGFR mutations. Ther Adv Med Oncol (2021) 13:1758835921992977. doi: 10.52389/ydls.v15iTA.733 33680095 PMC7900790

[27] National Drug System Development Committee. Announcement of the national drug system development committee. re: National list of essential drugs B.E. 2563. Thai Government Gazette (2020). Available at: https://specialty.mims.com/topic/thailand-national-list-of-essential-medicines--nlem-#resources

