# Supplementary figures and images for: Healthcare coverage affects survival of EGFR-mutant Thai lung cancer patients
Source: Front Oncol. 2023 Feb 21;13:1047644. doi: 10.3389/fonc.2023.1047644 (PMC9989298; doi:10.3389/fonc.2023.1047644)

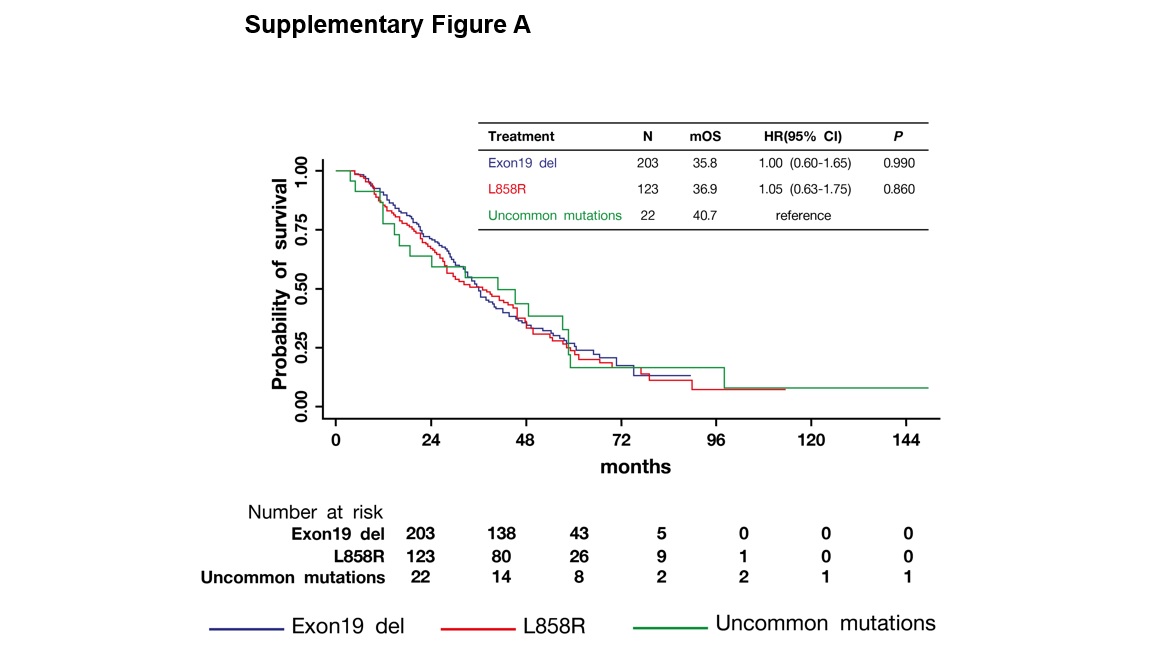

Supplement: Supplementary Figure A — Overall survival by EGFR mutation subtype. mOS = median overall survival; HR = hazard ratio; CI = confidence interval. [file Image_1.jpeg]

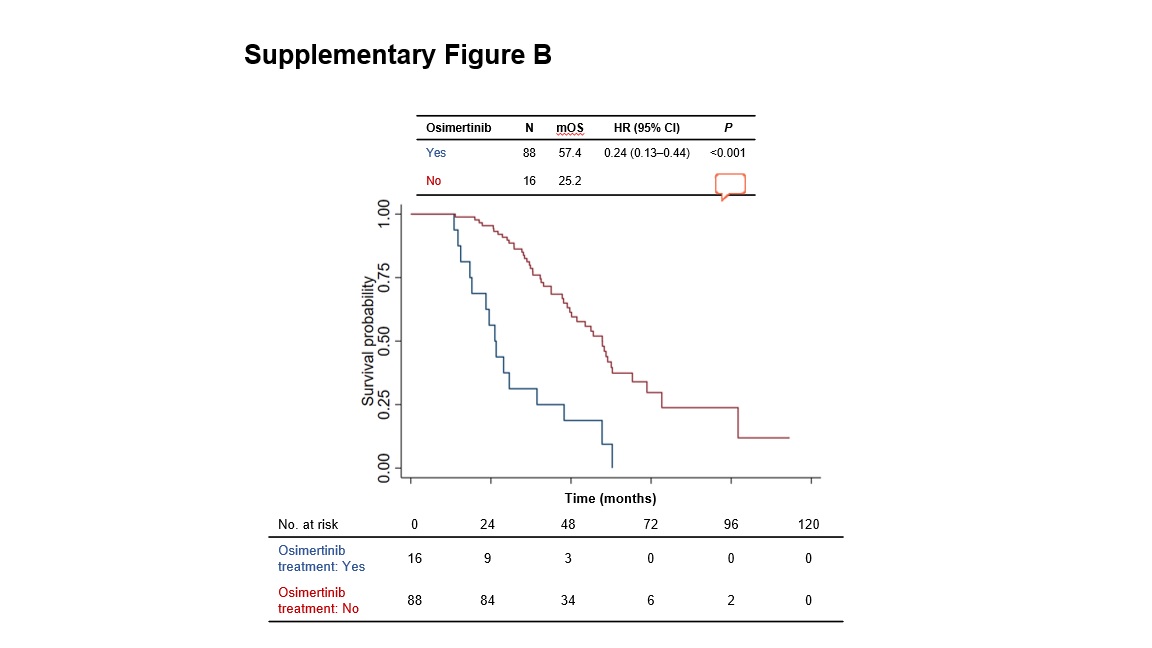

Supplement: Supplementary Figure B — Overall survival in patients with acquired T790M mutation with and without osimertinib treatment. mOS = median overall survival; HR = hazard ratio; CI = confidence interval. [file Image_2.jpeg]
