# Supplementary material for: Healthcare coverage affects survival of EGFR-mutant Thai lung cancer patients
Source: Front Oncol. 2023 Feb 21;13:1047644. doi: 10.3389/fonc.2023.1047644 (PMC9989298; doi:10.3389/fonc.2023.1047644)
Supplement: Supplementary file 3 [file Table_1.docx]

**Supplementary Table A: *EGFR* mutation status and subtypes identified**

| ***EGFR* mutation status** | **N=750** |
| --- | --- |
| No *EGFR* mutation | 328 (43.7) |
| *EGFR* mutation | 422 (56.3) |
| Exon 19 deletion (Del19) | 234 (55.2) |
| L858R | 138 (32.7) |
| G719X | 11 (2.1) |
| L861Q | 5 (1.2) |
| Exon 20 insertion | 11 (2.6) |
| Del19 + L858R | 3 (0.4) |
| Del19 + L861Q | 1 (0.1) |
| Del19 + S768I | 1 (0.1) |
| Del19 + T790M | 3 (0.4) |
| L858R + exon 20 insertion | 2 (0.1) |
| L858R + S768I | 2 (0.3) |
| L858R + T790M | 5 (0.7) |
| G719X + S768I | 5 (0.5) |
| L861Q + T790M | 1 (0.1) |

n (%) unless otherwise stated.

**Supplementary** **Table B: Systemic therapy for patients with advanced/recurrent *EGFR*m-positive NSCLC**

| **Systemic therapy received** | **First-line treatment**  **N=750** | **Second-line treatment**  **N=646** | **Third-line treatment**  **N=456** |
| --- | --- | --- | --- |
| **EGFR-TKIs** | **208 (27.7)** | **180 (27.9)** | **103 (22.6)** |
| Erlotinib | 63 (8.4) | 57 (8.8) | 20 (4.4) |
| Gefitinib | 111 (14.8) | 65 (10.0) | 38 (8.3) |
| Afatinib | 20 (2.7) | 14 (2.2) | 12 (2.6) |
| Osimertinib | 7 (0.9) | 44 (6.8) | 32 (7.0) |
| EGFR-TKIs in clinical trials | 7 (0.9) | 0 (0.0) | 1 (0.2) |
|  |  |  |  |
| **Chemotherapy or other** | **438 (58.4)** | **276 (42.7)** | **178 (39)** |
| Doublet cisplatin-based CMT | 12 (1.5) | 3 (0.5) | 1 (0.2) |
| Doublet carboplatin-based CMT | 360 (43.8) | 77 (11.9) | 49 (10.8) |
| Single agent CMT | 61 (7.4) | 183 (28.3) | 110 (24.1) |
| CMT + anti-VEGF | 2 (0.2) | 6 (0.9) | 3 (0.7) |
| Checkpoint inhibitors | 3 (0.4) | 6 (0.9) | 15 (3.3) |
| Others | 0 (0.0) | 1 (0.2) | 0 (0.0) |
|  |  |  |  |
| **No systemic therapy** | **104 (13.9)** | **190 (29.4)** | **175 (38.4)** |

n (%) unless otherwise stated.
CMT = Chemotherapy; EGFR-TKI = Epidermal growth factor receptor-tyrosine kinase inhibitor; *EGFR*m = positive for *EGFR* activating mutation; VEGF = vascular endothelial growth factor

**Supplementary Table C: Time to treatment failure (TTF) according to *EGFR* mutation subtype in *EGFR*m-positive patients treated with EGFR-TKIs (N=348)**

| ***EGFR* mutation subtype** | **N** | **TTF  (months)** | **HR (95%CI)** | ***P*^†^** |
| --- | --- | --- | --- | --- |
| Exon 19 deletion | 188 | 13.5 | reference |  |
| L858R | 111 | 13.1 | 1.25 (0.97–1.61) | 0.079 |
| Single uncommon mutation | 12 | 9.1 | 1.36 (0.74–2.51) | 0.326 |
| Double mutation, T790M+ | 8 | 7.6 | 3.22 (1.57–6.60) | 0.001* |
| Double mutation, T790M− | 9 | 15.0 | 0.70 (0.34–1.43) | 0.328 |

**^†^** P-values were calculated from Cox proportional hazards model.
^*^ P-values <0.05 were considered statistically significant.
Abbreviations: EGFR-TKI = epidermal growth factor receptor-tyrosine kinase inhibitor; CI = confidence interval; HR = hazard ratio; TTF, time to treatment failure
